# Fermented Foods: Raw Materials, Microorganisms, Emerging Technologies, and Novel Products

**DOI:** 10.3390/foods14213775

**Published:** 2025-11-04

**Authors:** Meilin Wang, Yongqi Yin

**Affiliations:** College of Food Science and Engineering, Yangzhou University, Yangzhou 225009, China

## 1. Introduction

Fermented foods constitute an integral component of the human diet, evolving from ancient preservation techniques to sophisticated nutritional matrices in modern food systems [1,2]. Contemporary fermentation serves not only to extend shelf life and impart distinctive sensory characteristics—from the umami depth of soy sauce to the complex flavor profiles of fermented dairy products—but also enhances the nutritional benefits of foods through improved nutrient bioavailability, probiotic physiological effects, and bioactive compound synthesis modulation [3]. Microbial fermentation enriches the nutritional and bioactive characteristics of food matrices through the biosynthesis of diverse metabolites, including bioactive peptides, phenolic compounds, organic acids, short-chain fatty acids (SCFAs), exopolysaccharides (EPSs), and essential vitamins. These metabolites exhibit significant physiological activities, including cardiovascular protective effects, enhanced lipid homeostasis, glycemic regulation, immunomodulation, neuroprotective mechanisms, and antioxidant capacity, establishing fermented foods as strategic components of functional nutrition [4]. In recent decades, fermented food research has entered a transformative phase, driven by consumer demand for sustainable, functional, and clean-label products, alongside scientific advances in microbial ecology, raw material valorization, and fermentation engineering [5,6,7]. This transformation presents critical research questions: How can novel fermentation strains be screened and utilized to enhance the nutritional properties of foods? How can low-value agricultural and industrial by-products be effectively converted into high-quality fermentation substrates to promote sustainable development in the food industry? How can traditional and emerging technologies be balanced to develop next-generation fermented foods? This Special Issue of *Foods*, titled “Fermented Foods: Raw Materials, Microorganisms, Emerging Technologies, and Novel Products,” presents six original research articles from seven research teams across three countries. These six pioneering studies provide novel insights into fermented foods, demonstrating current progress and future directions in this dynamic field, contributing to the sustainable development of fermented foods.

## 2. Microbial Resources: From Isolation to Functional Application

Microorganisms constitute the cornerstone of fermentation processes, and the isolation and identification of novel fermentative strains possessing beneficial characteristics—including antimicrobial properties, probiotic potential, or bioactive metabolite synthesis capabilities—remain pivotal research objectives [8,9]. Three manuscripts in this Special Issue focus on fermentative strain development, emphasizing their roles in enhancing food safety and bioactivity. Guan et al. (Contribution 1) successfully isolated and taxonomically identified *Lacticaseibacillus rhamnosus* YT from human intestinal sources, comprehensively evaluating the antimicrobial properties of both its cell-free supernatant and cellular components. Their investigation demonstrated that the strain’s cell-free culture supernatant exhibited growth phase-dependent, enzyme-insensitive, pH-responsive, and thermostable antimicrobial efficacy against both Gram-positive and Gram-negative pathogens, including *Bacillus subtilis* and *Salmonella enterica ser Enteritidis*, while the cellular fraction displayed broad-spectrum inhibitory effects through thermosensitive cell surface-associated compounds characterized by heat sensitivity and specific enzyme susceptibility. This dual antimicrobial mechanism—involving both extracellular metabolites and cellular components—establishes *Lacticaseibacillus rhamnosus* YT’s potential as an alternative antimicrobial agent in fermented food safety applications, addressing industrial demand for safe, naturally derived alternatives to chemical antimicrobials. Song et al. (Contribution 2) employed *Cordyceps militaris*, a nutritional fungus rich in cordycepin and bioactive polysaccharides, as a monoculture fermentation starter for functional soy sauce production, replacing conventional multi-strain inoculation protocols typically utilizing *Aspergillus oryzae*. Through optimized fermentation processes, they produced a novel soy sauce containing 1.14 ± 0.05 g/100 mL amino acid nitrogen and 16.88 ± 0.47 mg/100 mL cordycepin. This soy sauce demonstrated enhanced umami characteristics and distinctive volatile compound profiles—including linoleic acid, palmitic acid methyl ester, and nicotinamide—compared to conventionally fermented products. This study validates the feasibility of utilizing medicinal fungi for developing functional fermented condiments, providing a paradigm for functional fermented food development. Jiang et al. (Contribution 3) screened lactic acid bacteria exhibiting potent 2,2-Diphenyl-1-picrylhydrazyl radical scavenging capacity, hydroxyl radical inhibition, and elevated superoxide dismutase activity. They systematically evaluated the impact of these antioxidant-competent lactic acid bacteria on bitterness, mouthfeel, and flavor profiles of highly hydrolyzed enzymatically defatted milk. Their findings revealed that *Lactiplantibacillus plantarum* 16 effectively ameliorated the bitter taste and flavor characteristics of enzymatically defatted milk while significantly enhancing antioxidant capacity. These three investigations collectively demonstrate the substantial potential of selected superior strains in elevating the quality and nutritional attributes of fermented foods.

## 3. Raw Material Valorization: From By-Products to Specialized Crops

The substrate composition of fermentation media fundamentally determines the nutritional and bioactive potential of fermented products, with contemporary research emphasizing two strategic approaches: valorization of agro-industrial waste streams and optimization of specialized raw materials for fermentation applications [10]. This Special Issue presents three studies advancing these research priorities. Guilherme et al. (Contribution 4) leveraged agro-industrial byproducts including sugarcane molasses, corn steep liquor, and residual yeast biomass as fermentation media for *Schizochytrium* sp. SR21. Through laboratory-scale optimization using central composite design, they achieved 1.12-fold, 1.72-fold, and 1.92-fold improvements in biomass yield, lipid accumulation, and total lipid productivity, respectively. Bioreactor scale-up to a 10 L stirred-tank configuration achieved 39.29 g/L biomass concentration, 14.98 g/L lipid content, and 32.83% docosahexaenoic acid composition in total lipids. This work established a sustainable circular bioeconomy framework for agro-industrial waste utilization while reducing ω-3 production costs. Chinese baijiu represents one of the world’s oldest distilled spirits, with wheat-based medium–high-temperature Daqu serving as the essential fermentation starter for baijiu production. However, the complex interactions between wheat cultivars, cultivation environments, and Daqu quality characteristics remain insufficiently understood. Zhou et al. (Contribution 5) evaluated three wheat varieties (B4361, Chuanmai 39, Lunuomai 3) harvested from different cultivation environments (Chongzhou, Mianzhu, Luzhou), revealing that (1) cultivation environment primarily influences wheat endophytic fungal communities (including *Stemphylium* and *Dioszegia*), while variety predominantly determines wheat physicochemical properties (protein and starch content); (2) key microbial taxa (*Pantoea*, *Aspergillus*, and *Stemphylium*) exhibit transmission from wheat substrate to Daqu; (3) wheat starch content and amino acid nitrogen concentration drive Daqu microbial community succession. This “cultivation environment–grain endophytes–Daqu microbiota” pathway establishes the foundation for developing wheat varieties specifically optimized for Daqu production and identifying optimal cultivation regions.

## 4. Emerging Technologies: From Process Optimization to Nutrient Fortification

Fermentation processes are crucial for the quality, processing efficiency, and bioactive properties of fermented foods [11]. Hypertensive patients are often advised to avoid high-sodium fermented foods such as pickles, olives, and sauerkraut, despite their high nutritional value, including rich content of monounsaturated fatty acids, polyphenols, and minerals. López-López et al. (Contribution 6) established an innovative mineral fortification protocol for traditional green Manzanilla Aloreña olives (a naturally processed variety) by supplementing a salt mixture of KCl, CaCl_2_, and MgCl_2_ during the final packaging stage post-fermentation, thereby achieving sodium reduction while enhancing mineral bioavailability. The pre-packaging desalination procedure achieved 61% sodium reduction while significantly enriching mineral content. This post-fermentation fortification strategy circumvents potential contamination risks associated with low NaCl concentrations during fermentation while meeting consumer preferences for low-sodium, micronutrient-enhanced fermented products, promoting the development of nutritionally enriched natural table olives and providing novel strategies for sodium reduction in the food industry. In this study, they also achieved predictive quantification of mineral concentrations and their contribution to Reference Daily Intake (RDI) values through Response Surface Methodology (RSM) modeling, with calcium supplementation achieving up to 70% RDI compliance and identifying optimal salt combinations for specific targets. Response surface methodology has broad applications in fermented food process optimization. Song et al. (Contribution 2) and Guilherme et al. (Contribution 4) similarly employed RSM optimization combined with bioreactor scale-up approaches to enhance fermentation bioprocessing—demonstrating the critical integration of statistical experimental design and bioprocess engineering in the commercial translation of laboratory-scale protocols. The 10 L bioreactor scale-up of *Schizosaccharomyces pombe* SR21 cultivation successfully addressed key bioprocess parameters, including dissolved oxygen management and substrate conversion efficiency, while Cordyceps militaris optimization achieved maximum protease activity and cordycepin biosynthesis.

## 5. Conclusions

In summary, this Special Issue underscores the vitality of fermented food research, with contributions spanning microbial resource mining, raw material valorization, technological innovation, and product development. We hope this Special Issue inspires further advances in fermented food science and contributes to a more sustainable, nutritious, and diverse food supply.
